# MiR-320a upregulation improves IL-1*β*-induced osteoarthritis via targeting the DAZAP1 and MAPK pathways

**DOI:** 10.1186/s13018-023-03984-2

**Published:** 2023-07-28

**Authors:** Jing Mao, Lei Zhang

**Affiliations:** 1 Department of Rheumatology and Immunology, Jingzhou First People’s Hospital, Yangtze University, Jingzhou, Hubei China; 2 Department of Dermatology, Jingzhou First People’s Hospital, Yangtze University, Jingzhou, Hubei China

**Keywords:** Osteoarthritis, miR-320a, IL-1*β*, DAZAP1, MAPK

## Abstract

**Purpose:**

Osteoarthritis (OA), a constant illness described by articular cartilage degeneration, usually manifested by joint pain and helpless development. Numerous literatures suggest that microRNAs play an important regulatory role in OA, yet the role of miR-320a in OA remains largely obscure.

**Materials and methods:**

To evaluate the expression of miR-320a mRNA, quantitative real-time polymerase chain reaction was used. Cell counting kit-8 assay, Edu staining, Annexin V-FITC/PI apoptosis detection assay, Caspases 3 staining, and trypan staining were conducted to monitor cell proliferation and apoptosis. Western blot was applied to examine DAZAP1 and ERK/JNK/MAPK associated protein expression. Luciferase reporter gene experiments were performed to confirm the relationships between miR-320a and DAZAP1. ELISA assay was adopted to analyze the secretion of inflammation cytokines IL-6, IL-8, and TNF-*α*.

**Results:**

In an in vitro osteoarthritis model caused by IL-1*β*, miR-320a expression was markedly reduced. Overexpression of miR-320a restored IL-1*β*-inhibited chondrocyte proliferation, induced apoptosis and inflammatory response. Mechanistically, miR-320a affected HC-A cell proliferation, apoptosis and inflammatory response by regulating DAZAPI. Meanwhile, the ERK/JNK/MAPK pathway is also involved in the regulatory role of miR-320a on OA.

**Conclusion:**

Our results show an important role for miR-320a and provide new therapeutic targets for avoiding and treating osteoarthritis.

## Introduction

Osteoarthritis (OA) is an ongoing arthropathy related to articular degeneration, appearing as articular ligament degeneration and bone hyperplasia, frequently found in the elderly [1, 2]. OA is normally appeared as joint torment and helpless development, and subchondral hardening, trabecular break, and cystic changes are displayed under X-beam assessment [3]. As of late, the OA frequency rate is on the ascent because of populace maturing. Studies have expressed that numerous fiery variables, like TNF-*α*, IL-6, and IL-1*β*, add to OA advancement. Outstandingly, the height of IL-1*β* works with the profiles of cyclooxygenase-2 (COX-2) and nitric oxide synthase (iNOS), hence facilitating OA advancement [4]. Thus, it is urgent to investigate into the particular system of OA and diminish the incendiary go between discharge in OA treatment.

MicroRNAs (miRNAs) are single-abandoned RNA atoms that are broad introduced in eukaryotes, containing around 17 to 25 nucleotides. MiRNAs control the outflow of target qualities by base blending to integral locales in the 3ʹ-untranslated district (3ʹ-UTR) of target mRNAs and lead to translational suppression or the debasement of the mRNAs [5]. An enormous number of past examinations have revealed that different miRNAs are involved in controlling the etiology of musculoskeletal disorders [6–8]. Lorenzo Giordano et al. [9] found that multiple miRNAs were involved in the process of tendon lesions by searching multiple databases, but the use of siRNA has a very important effect on rheumatoid arthritis, tendon regeneration, and healing [10, 11]. Iliopoulos et al. [12] checked the declarations of 365 miRNAs in particular ligament acquired from OA patients and observed that 16 miRNAs were differentially communicated during OA, and miRNA is involved in the physiological and pathological mechanism of osteoarthritis, and because of the regulation of miRNA gene expression, it can be used as a marker miRNA for the diagnosis of osteoarthritis [13]. In OA chondrocytes, for example, miR483, miR-22, and miR-377 expression was upregulated, while miR-140, miR-29a, and miR-25 expression was downregulated [12]. Besides, miR-148a has been found to go about as a possibly illness adjusting compound in OA, as miR-148a overexpression advances hyaline ligament production [14]. MiR-33a has been recognized as an objective for enhancing the OA aggregate by controlling cholesterol blend and cholesterol effluxrelated genes [15].

MiR-320a has a place with the miR-320 family and assumes a vital part in numerous sicknesses, for example, Waldenstrom macroglobulinemia (WM), cerebral ischemia and different diseases [16–19]. In any case, the job of miR-320a in the pathogenesis of OA has not yet been uncovered [20]. In this article, the outflow of miR-320a and its objective quality was examined in OA chondrocytes. Moreover, the impacts of dysregulation of miR-320a and its objective quality on interleukin 1 beta (IL-1*β*)-prompted network still up in the air in vitro. This study may furnish us with a fundamental comprehension of miR-320a on OA.

## Materials and methods

### Cell culture and stimulation

The human chondrogenic HC-A cells (ATCC, USA) were cultured in DMEM/F12 medium (Sigma-Aldrich, St. Louis, MO) supplemented with 5% FBS (Gibco, Grand Island, NY), 100 U/mL penicillin (Sigma-Aldrich), and 100 μg/mL streptomycin (Sigma-Aldrich) in a humidified incubator with 95% air and 5% CO_2_ at 37 °C. The cells were routinely cultured in in 6-well plates (Corning, New York, NY) with a density of 6 × 10^4^/well. The recombinant IL-1*β* with purity greater than 98% (HPLC) was purchased from Sigma-Aldrich. Cells were treated by 5 μg/mL IL-1*β* for 24 h.

### Cell transfection

The miR-320a mimic was synthesized by GenePharma Co (Shanghai, China). The full-length wide-type DAZAP1 sequences were inserted into the pEX-2 plasmid (GenePharma). An empty pEX-2 plasmid was transfected as a negative control. siRNA specific for DAZAP1 was constructed into U6/Neo plasmid (GenePharma). An empty U6/Neo plasmid with non-targeting sequences was transfected as a negative control. Cell transfections were conducted using Lipofectamine 3000 reagent (Invitrogen) under non-serum condition.

### CCK-8 assay

The transfected HC-A cells (5 × 10^3^) in 96-well plate were treated by IL-1*β* for 24 h. After one more 24 h of brooding at new culture medium, cell suitability was surveyed by utilizing CCK-8 (Dojindo Molecular Technologies, Kyushu, Japan). Momentarily, the way of life medium was taken out, and the cells were washed two times with phosphate cushion saline (PBS). 10 μL CCK-8 arrangement was added, and the plates were hatched for 1 h at 37 °C. The absorbance was estimated at 450 nm utilizing a Microplate Reader (Bio-Rad, Hercules, CA).

### Apoptosis assay

After transfection and IL-1 administration, the HC-A cells (5 × 10^5^) in 6-well plates were examined for apoptosis using the Annexin V-FITC/PI apoptosis detection kit (Invitrogen, Carlsbad, CA). In the presence of 50 g/mL RNase A, 1 × 10^5^ cells from each sample were washed twice with PBS and stained with Annexin V-FITC and PI according to the manufacturer's protocol (Sigma-Aldrich). The FACS can discriminate between apoptotic and non-apoptotic cells (Beckman Coulter, Fullerton, CA). FlowJo software was used to quantify the data (Tree Star Inc. Ashland, OR).

### Trypan blue staining assay

After cell transfection, samples from different groups were seeded at 5 × 104 cells per well in the 24-well plates at 37 °C for 48 h. Samples were then washed, trypsinized, and treated with trypan blue dye (Beyotime, Shanghai, China). Samples were counted in cell counting chamber.

### qRT-PCR

After transfection and IL-1*β* treatment, qRT-PCR was used to assess the mRNA levels of miR-320a in HC-A cells. The RNA pure Rapid Extraction Kit was used to extract total RNA from HC-A cells (Bioteke Corporation, Beijing, China). The SuperScriptTM IV First-Strand Synthesis System was used to transcribe the cDNA (Invitrogen). The mRNA and miRNA expressions were detected using SYBRTM Green PCR Master Mix (Applied Biosystems, Foster City, CA) and mirVanaTM qRT-PCR miRNA Detection kit (Invitrogen). Internal controls included GAPDH and U6.

### ELISA

After transfection and IL-1 treatment, the culture supernatant was collected from HC-A cells (5 × 10^4^) in 24-well plates. ELISA kits (R&D Systems, Abingdon, UK) were used to measure the quantities of IL-6, IL-8, and TNF-*α* in the culture supernatant according to the manufacturer's procedure.

### Western blot

All out protein in HC-A cells after important transfection and treatment were removed utilizing RIPA lysis cushion (Beyotime, Shanghai, China). The virtue of proteins was measured by the BCA™ Protein Assay Kit (Pierce, Appleton, WI). Western blotch framework was laid out utilizing a Bio-Rad Bis–Tris Gel framework (Bio-Rad Laboratories, Hercules, CA). Proteins in equivalent fixation were electrophoresed in PAGE-SDS and moved onto PDVF films (Millipore, Bedford, MA). Subsequent to impeding in the 5% obstructing cradle (Beyotime), the films were brooded with essential antibodies which were ready in 5% hindering support at a weakening of 1:1, 000. After hatching with essential antibodies at 4 °C short-term, the PDVF films were brooded with auxiliary immunizer for 1 h at room temperature. Signs of the groups were caught utilizing Bio-Rad ChemiDocTM XRS framework (Bio-Rad). The power of the groups was measured utilizing Image Lab™ Software (Bio-Rad). The intensity of the bands was quantified using Image Lab™ Software (Bio-Rad).

### Statistical analysis

Unless part of the data from Western blot analysis, all other data are presented as the mean ± SD. Statistics were analyzed by the one-way analysis of variance (ANOVA) in SPSS 19.0 statistical software (SPSS Inc., Chicago, IL). A p-value of lower than 0.05 was considered as a significant result.

## Results

### *Establishment of an *in vitro* model of osteoarthritis*

**Fig. 1:**
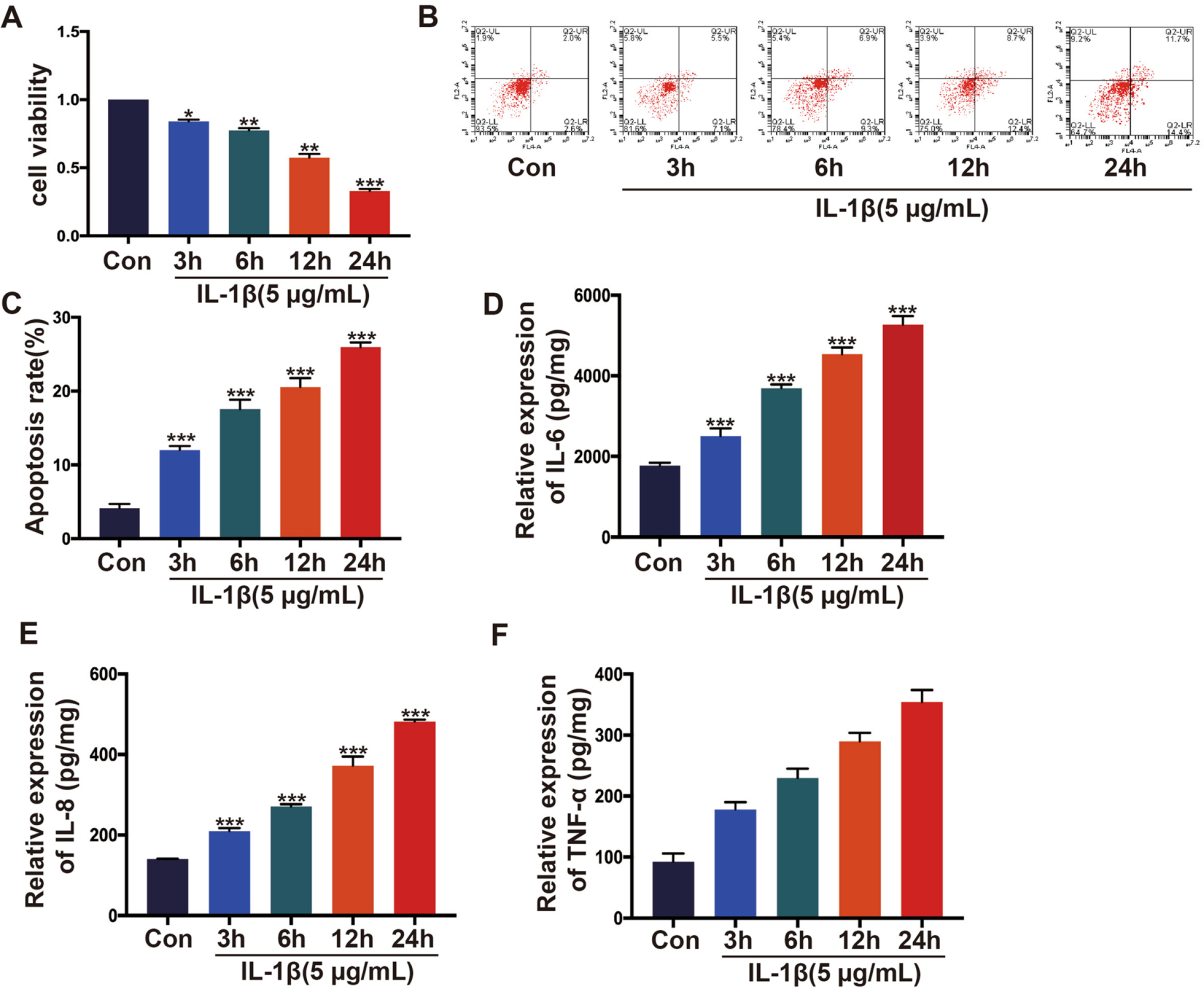
Construction of OA model in vitro. IL-1*β* was induced to construct OA model. IL-1*β* (5 µg/mL) was adopted to treat HC-A human chondrocyte. **A** CCK-8 tested cell viability. **B**, **C** Annexin V/PI assay tested cell apoptosis. **D**–**F** The expression of IL-6, IL-8, and TNF-*α* was monitored by ELISA. *p < 0.05,**p < 0.01,***p < 0.001. *N *= 3

The effect of IL-1*β* on chondrocytes was surveyed by a CCK-8 kit. Cells were incubated with IL-1*β* at a concentration of 5 µg/mL for 3 h, 6 h, 12 h and 24 h, respectively. As shown in Fig. 1A, HC-A cells had a time-dependent effect on IL-1*β*. Compared with the control group, with the continuous increase in IL-1*β* stimulation time, the cell viability decreased continuously, and the IL-1*β* stimulation time was 12 h; the percentage of cell viability was 50%. Therefore, IL-1*β* at a concentration of 5 µg/mL for 12 h was selected for subsequent experiments. Next, we detected the apoptosis rate of HC-A cells after stimulation with IL-1*β* by the Annexin V. Figure 1B and C shows that with the prolongation of IL-1*β* stimulation time, the apoptosis rate of HC-A cells was higher. The Elisa test detected the expression of inflammatory factors TNF-*α*, IL-6, and IL-8, and Fig. 1D–F indicated that IL-1*β* stimulation can result in upregulation of TNF-*α*, IL-6, and IL-8 expression, with a time-dependent relationship.

### *The role of miR-320a in osteoarthritis-induced cell damage *in vitro

**Fig. 2:**
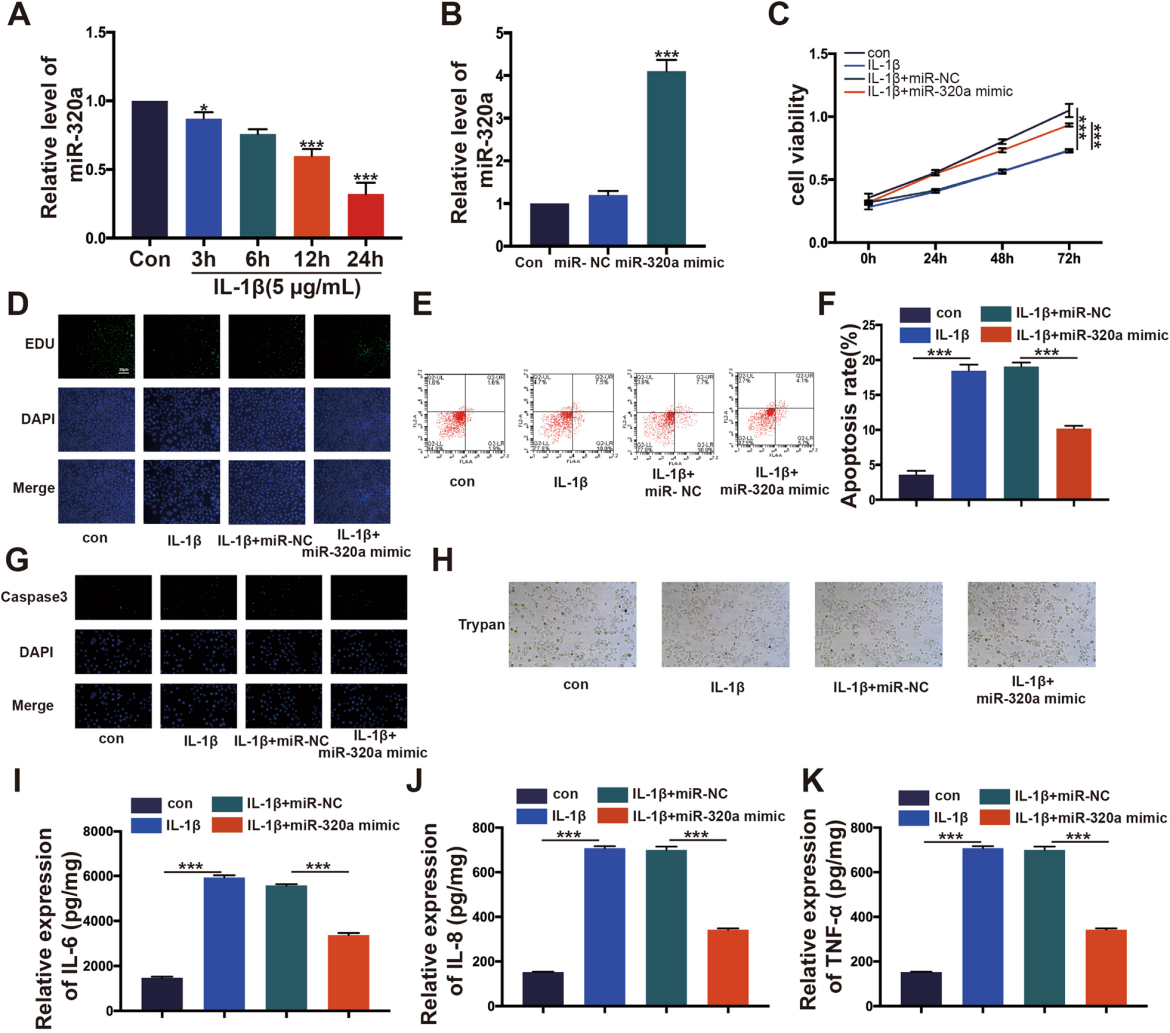
Overexpressing miR-320a aggravated IL-1*β*-mediated chondrocyte apoptosis and inflammation. MiR-320a mimics was transferred into HC-A cells, respectively. **A** The miR-320a expression in OA model was verified by RT-PCR, *N *= 3. **B** The miR-320a profile was measured by RT-PCR. **C** CCK-8 tested cell viability. **D** The EDU expression in HC-A cells was verified by Immunofluorescence. **E**–**H** Cell apoptosis was tested by Annexin V/PI assay, Immunofluorescence, and Trypan. **I**–**K** The expression of IL-6, IL-8, and TNF-*α* was monitored by ELISA. *p < 0.05,**p < 0.01,***p < 0.001. *N *= 3

In the IL-1*β* stimulated osteoarthritis model, we found that the expression of miR-320 was significantly downregulated in a time-dependent manner is shown in Fig. 2A. To explore the role of miR-320a in an in vitro osteoarthritis model, we overexpressed miR-320a in HC-A cells. Significantly enhanced expression of miR-320a was observed in cells transfected with miR-320a as compared to scramble cells and is shown in Fig. 2B (*p *< 0.01). A decreased cell viability was observed in cells transfected with miR-320a compared to scramble cells (*p *< 0.05) (Fig. 2C). The proliferation rate of cells overexpressing miR-320a increased after IL-1*β* stimulation compared to normal cells and was comparable to the control group (*p *< 0.05) (Fig. 2D). After IL-1*β* stimulation, apoptosis was significantly increased in cells transfected with miR-320a-mimic compared to miR-320a mimic negative control (*p *< 0.05), as observed by Annexin V-FITC and PI, NucView™Caspase-3 kit and Trypan blue staining assay (Fig. 2E–H). In addition, an ELISA assay revealed that cells transfected with miR-320a-mimic released less IL-6, IL-8, and TNF-*α* than miR-320a mimic negative control cells after IL-1*β* stimulation (p 0.05) (Fig. 2I–K). The release of IL-6, IL-8, and TNF-*α* was influenced by miR-320a, as expected.

### DAZAP1 is a target of miR-320a and mediates OA pathogenesis

**Fig. 3:**
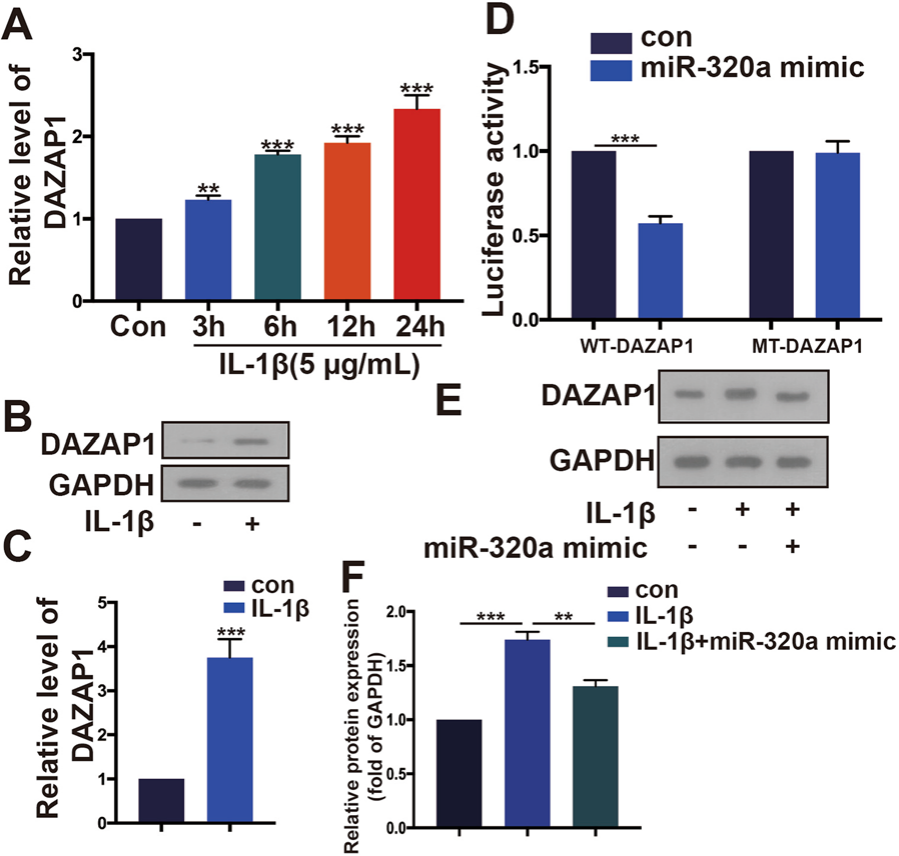
MiR-320a targeted DAZAP1. **A–C** The expression of DAZAP1 in OA model by RT-PCR and WB, respectively. *N *= 3. **D** The luciferase activity in HC-A cells after miR-320a mimics or si- DAZAP1 was detected by luciferase assay. **E**, **F** The expression of DAZAP1 in OA model after miR-320a mimics by WB. *p < 0.05,**p < 0.01,***p < 0.001. *N *= 3

To determine whether DAZAP1 is a target of miR-320a, we first examined the expression of DAZAP1 after IL-1*β* stimulation by qRT-PCR and Western blotting assays. As shown in Fig. 3A–C, DAZAP1 was upregulated in cells treated with IL-1*β* as compared to the control cells, with a time-dependent relationship. Compared with mt-DAZAP1, the addition of miR-320a mimic significantly decreased the luciferase activity in the wt-DAZAP1 group, while in mt-DAZAP1, the addition of miR-320a mimic hardly changed the luciferase activity. This indicates that there is a targeting relationship between miR-320a and DAZAP1 (Fig. 3D). Western blot analysis indicated that DAZAP1 protein level was downregulated by miR-320a mimic with IL-1*β* stimulation (Fig. 3E and F).

Regulation and mechanism of miR-320a/DAZAP1 on IL-1*β*-induced HC-A cell injury.

**Fig. 4:**
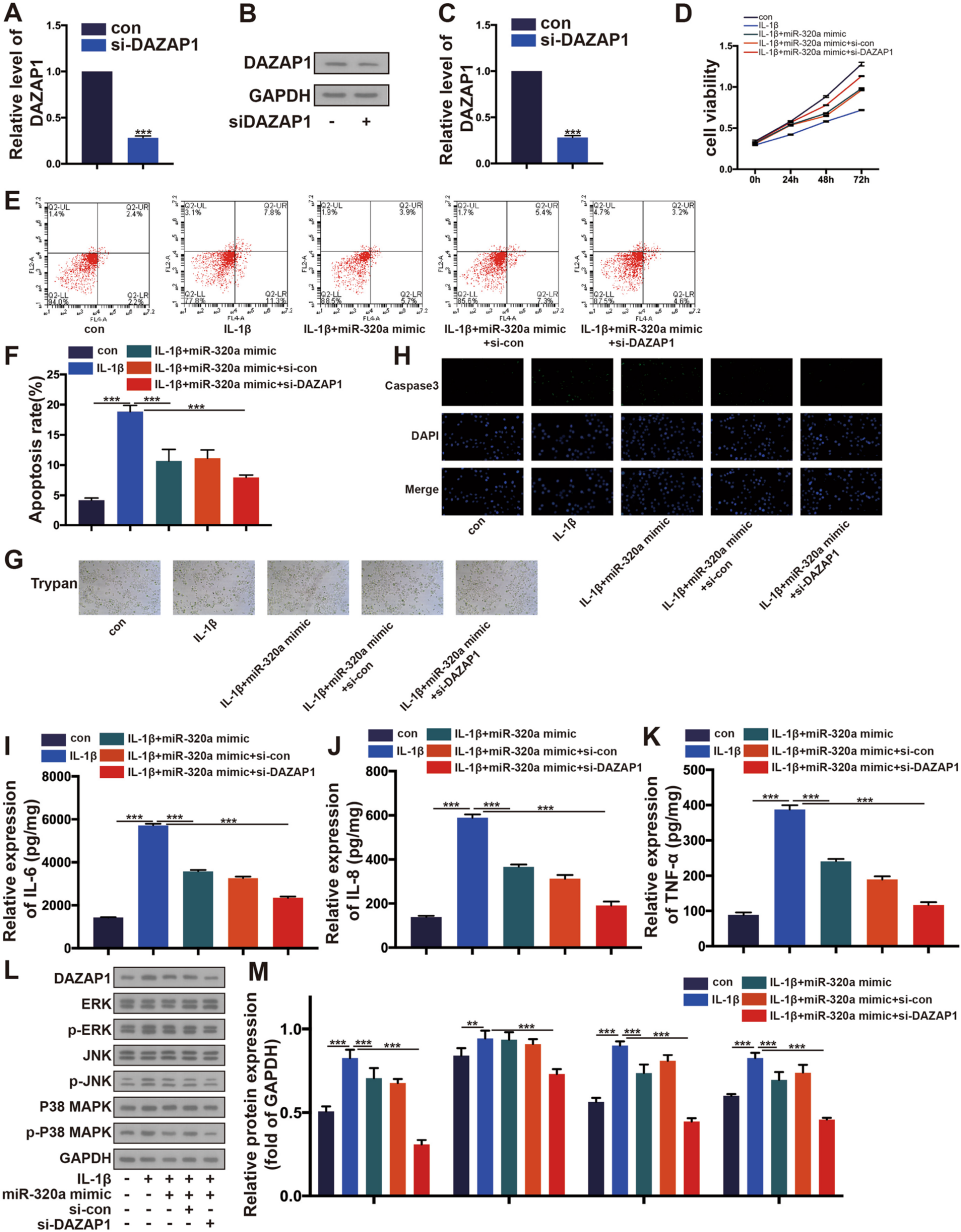
The miR-320a/DAZAP1/MAPK could regulate IL-1*β*-mediated chondrocyte in vitro. **A**–**C** RT-PCR and WB assay detection after HC-A cells being treated with si-DAZAP1. **D** CCK-8 tested cell viability. **E**–**H** Cell apoptosis was tested by Annexin V/PI assay, Immunofluorescence, and Trypan. **I**–**K** The expression of IL-6, IL-8, and TNF-*α* was monitored by ELISA. **L**, **M** The expression of DAZAP1, p-ERK, p-JNK, p-P38 MAPK in OA model after miR-320a mimics or si-DAZAP1 by WB. *p < 0.05,**p < 0.01,***p < 0.001. *N *= 3

To explore the regulatory role of miR-320a/DAZAP1 on IL-1*β*-induced HC-A cell injury, we first silenced DAZAP1 in HC-A cells. Detection of DAZAP1 silencing efficiency using qRT-PCR and Western blotting assays Fig. 4–C. A upregulated viability was observed in the cells overexpressing miR-320a and si-DAZAP1 with IL-1*β* compared to the cells overexpressing miR-320a (*p *< 0.05) (Fig. 4D). After silenced DAZAP1, apoptosis was significantly decreased in cells with miR-320a-mimic and IL-1*β* compared to cells with miR-320a mimic and IL-1*β* group (*p *< 0.05), as observed by Annexin V-FITC and PI, NucView™Caspase-3 kit and Trypan blue staining assay (Fig. 4E–H). Also, reduced release of IL-6, IL-8, and TNF-*α* was observed in cells transfected with miR-320a mimic and si-DAZAP1 compared to cells with miR-320a mimic after IL-1*β* stimulated (*p *< 0.05) (Fig. 4I–K). A significant decrease in p-ERK, p-JNK, p-p38 MAPK expression was observed in cells transfected with miR-320a mimic group and transfected with miR-320a mimic and si-DAZAP1 group compared to cells with control cells and IL-1*β* stimulated group (Fig. 4L and M).

## Discussion

OA is regularly connected with numerous gamble factors, most strikingly age, joint injury, adjusted biomechanics, and heftiness [21, 22]. There are logical reports, recommending that pathologic changes connected with OA share a typical last pathway and hence, work to sustain joint annihilation and possible disappointment. It was accounted for that IL-1*β* was associated with the pathogenesis of OA that it could fundamentally decrease chondrocytes multiplication, prompt cell demise, and speed up aggravation [23, 24]. This was likewise affirmed in the current review that IL-1*β* decreased HC-A cells practicality, initiated apoptosis, and expanded the arrival of favorable to fiery cytokines, similar to TNF-*α*, IL-6 and IL-8. This information showed that apoptotic and fiery injury was fundamentally instigated by IL-1*β* in HC-A cells.

We additionally showed that miR-320a was underexpressed in light of IL-1*β* treatment, implying that miR-320a might be a potential regulator in the pathogenesis of OA. All the more significantly, miR-320a underexpression constricted IL-1*β*-incited harm in HC-A cells, as confirmed by expanded feasibility, diminished apoptosis, and stifled proinflammatory cytokine discharge. Late examinations have detailed that different miRNAs are involved in the pathogenesis of OA. Be that as it may, the job of miR-320a in OA has not yet been uncovered. Here, we revealed that miR-320a was decreased during OA, inferring that miR-320a may be an expected controller in the pathogenesis of OA. DAZAP1 was an immediate objective of miR-320a and was adversely managed by miR-320a. Of note, DAZAP1 and MAPK signaling pathway may be associated with miR-320a-interceded IL-1*β*-initiated framework debasement factors in OA chondrocytes.

DAZAP1 was first discovered as a protein that interacts with DAZ and DAZL (DAZ-like protein), which are structurally related [25]. In germ cells, DAZ and DAZL, as well as another family member known as BOULE, are all highly expressed. Humans, big apes, and Old World monkeys all have the DAZ gene on their Y chromosomes [26–28], and it is commonly eliminated in male sterility [29]. Members of this family have been shown to play a function in protein translation stimulation. BOULE, for example, is required for the translation of the mRNA encoding Twine, a Cdc25-like phosphatase implicated in meiosis control in Drosophil [30]. DAZ and DAZL have also been discovered to be linked to polyribosomes that are actively involved in protein production [31]. In the present study, we found that DAZAP1 was a target gene of miR-320a. Besides, the protective actions of miR-320a suppression on IL-1*β*-induced cell injury were partially eliminated by DAZAP1 silencing. Our data indicated that miR-320a suppression alleviated inflammatory injury induced by IL-1*β* possibly via targeting DAZAP1 in HC-A cells.

The pathogenesis of OA involves multiple signaling pathways, among which MAPK signaling pathways play significant roles in cartilage damage [19, 32, 33]. Mitogen-activated protein kinases (MAPKs) are crucial regulators of cellular pathology and physiology and include ERK, p38, and JNK MAPK subfamilies [34–36]. Recent studies have shown that MAPK plays a crucial role in chondrogenic differentiation [37, 38]. The importance of p38/ERK MAPK and p65/NF-B signaling in macrophage activation and cartilage degradation, which promotes the pathogenesis and development of OA, is becoming clearer [39, 40]. A recent study found that activated ERK, p38, and JNK signaling pathways increased the production of IL-6 and TNF-*α* in human synovial fibroblasts [41]. Inhibition of the p38 MAPK signaling pathway decreases apoptosis in human OA chondrocytes, according to Kim et al. [42]. Here, we found that IL-1*β*-induced MAPK pathway activation was attenuated by overexpression of miR-320a and silencing of DAZAP1, suggesting that miR-320a/DAZAP1 protects HC-A by regulating this pathway.

To conclude, our findings in this study demonstrated a protective function of miR-320a overexpression on IL-1*β* injured HC-A cells. The protective actions might be released by targeting DAZAP1 and modulation of MAPK pathways. This study provides a novel insight into the role of miR-320a in IL-1*β* induced injury in OA. However, some limitations should be noted. First of all, this paper only carried out cell experiments in vitro to prove the effect of miRNA on osteoarthritis and did not carry out animal experiments in vivo. This may require us to do further research in this field, laying a foundation for the clinical application of miRNA transformation. In addition, the pathogenesis of osteoarthritis involves multiple signaling pathways, and we only studied one of them. In subsequent experiments, we will choose more signaling pathways. In conclusion, miRNA has great potential for the treatment of osteoarthritis, but so far, before transforming into clinical applications, we must first use miRNA as a therapeutic agent for osteoarthritis.

## Data Availability

The datasets generated during and/or analyzed during the current study are available from the corresponding author on reasonable request.
